# Estimation of Seasonal Correction Factors for Indoor Radon Concentrations in Korea

**DOI:** 10.3390/ijerph15102251

**Published:** 2018-10-15

**Authors:** Ji Hyun Park, Cheol Min Lee, Hyun Young Lee, Dae Ryong Kang

**Affiliations:** 1 Department of Mathematics, Ajou University, Suwon 16490, Korea; jhn1105@gmail.com; 2 Department of Chemical and Biological Engineering, SeoKyeong University, Seoul 02713, Korea; cheolminlee1@gmail.com; 3 Department of Statistics, Clinical Trial Center, Ajou University Medical Center, Suwon 16499, Korea; ajoustat@gmail.com; 4 Center of Biomedical Data Science/Institute of Genomic Cohort, Yonsei University Wonju College of Medicine, Wonju 26426, Korea

**Keywords:** indoor radon, seasonal variation, outdoor temperature, wind speed, mean annual radon concentrations, annual effective dose, Korea

## Abstract

Long-term exposure to high radon concentration exerts pathological effects and elicits changes in respiratory function, increasing an individual’s risk of developing lung cancer. In health risk assessment of indoor radon, consideration of long-term exposure thereto is necessary to identify a relationship between indoor radon exposure and lung cancer. However, measuring long-term indoor radon concentration can be difficult, and a statistical model for predicting mean annual indoor radon concentrations may be readily applicable. We investigated the predictability of mean annual radon concentrations using national data on indoor radon concentrations throughout the spring, summer, fall, and winter seasons in Korea. Indoor radon concentrations in Korea were highest in the winter and lowest in the summer. We derived seasonal correction and seasonal adjustment factors for each season based on the method proposed by previous study. However, these factors may not be readily applicable unless measured in a specific season. In this paper, we separate seasonal correction factors for each month of the year (new correction factors) based on correlations between indoor radon and meteorological factors according to housing type. To evaluate the correction factors, we assessed differences between estimated and measured mean annual radon concentrations. Roughly 97% of the estimated values were within ±40 Bq/m^3^ of actual measured values in detached houses, and roughly 85–87% of the estimated values were within ±40 Bq/m^3^ of the measured values in other residences. In most cases, the seasonal correction factors and the new correction factors had slightly better agreement than the seasonal adjustment factor. For predicting mean annual radon concentrations, the seasonal correction factors or seasonal adjustment factors can be of use when actual measurements of indoor radon concentrations for a specific season are available. Otherwise, the new correction factors may be more readily applicable.

## 1. Introduction

Radon is a major component of natural radiation that causes lung cancer, the second leading cause after smoking [1]. Radon in soil, building materials, outdoor air, and underground water enters indoors via various transfer mechanisms, and currently, many people spend most of their time in an indoor environment. Therefore, it is important to assess the health effects stemming from exposure to radon indoors. The health risk associated with exposure to radon indoors depends on the concentration thereof, the length of exposure, and indoor ventilation [2,3,4,5]. In health risk assessment of indoor radon, long-term exposure thereto must be considered to identify a relationship between indoor radon exposure and lung cancer.

According to an United Nations Scientific Committee on the Effects of Atomic Radiation (UNSCEAR) report [6], annual effective doses of radon are to be calculated as follows:

(1) E=Q×F×T×K

where *Q* is the mean annual radon concentration, *F* is the equilibrium factor between indoor radon and its decay product, *T* is the annual residential time, and *K* is the dose conversion coefficient. Therefrom, the annual effective dose can be assessed using annual radon concentrations and the annual lengths of time spent in a particular residence for individual occupants. In Korea, the official method designated by the Korean Ministry of Environment for measuring indoor radon concentrations utilizes the average concentration thereof over 3 months. However, as indoor radon concentrations can vary seasonally, an average over 3 months may not accurately represent mean annual radon concentrations.

Several countries have studied seasonal variations in indoor radon concentrations, and have reported similar patterns, with high concentrations in the winter and low concentrations in the summer. Moreover, researchers have established seasonal adjustment models, and derived seasonal correction factors for health risk assessment and management of indoor radon. Wrixon et al. [7] derived the first seasonal correction factors based on two consecutive measurements at 6-month intervals at 2300 residences in the UK. Thereafter, Pinel et al. [8] developed a model for determining seasonal correction factors for all 12 months of the year based on two 6-month measurements collected in the UK. In Canada, Krewski et al. [9] developed a simple multiplicative model for radon concentrations in residences based on data from a study conducted in Winnipeg, Canada. Additionally, Denman et al. [10] and Miles et al. [11] derived seasonal correction factors based on measurements at 3-month intervals in the UK. Additionally, in a recent study, Daraktchieva [12] showed a strong negative correlation between modeled indoor radon data and outdoor temperatures. With this relationship, new correction factors have been obtained. Meanwhile, studies in other countries, including France, Ireland, and Poland, have also derived and utilized correction factors [13,14,15].

For Korea, Lee et al. [16] calculated seasonal correction factors using the method proposed by Pinel et al. [8] and measurements at 3-month intervals conducted by the Korea Institute of Nuclear Safety (KINS) and the National Institute of Environmental Research (NIER). However, these correction factors might not be generally applicable if the measurements were taken over two or more seasons. Accordingly, we suggest that seasonal correction factors should be divided into separate factors for each month of the year. Thus, in this study, we derived seasonal correction and seasonal adjustment factors using the method proposed by Pinel et al. [8] based on data from surveys by the KINS and NIER. In addition, in the development of a model for predicting annual radon concentrations, we considered meteorological factors and separate seasonal correction factors for each month of the year (new correction factors). Finally, we compared the accuracies of 1-year predictions of annual indoor radon concentrations based on the new correction factors and those based on seasonal correction factors calculated using the method proposed by Pinel et al. [8].

## 2. Materials and Methods

### 2.1. Seasonal Variation of Indoor Radon Concentrations

Indoor radon concentrations at 3893 residences were measured during the spring (March–May), summer (June–August), fall (September–November), and winter (December–February) in 2005 by the KINS. In a later survey, indoor radon concentrations at 1154 residences were measured during the four seasons in 2010–2011 by the NIER. The two surveys were carried out using alpha track detectors, and included information on the type of residence and region.

Only dwellings that were monitored in all four seasons were used in this study. In addition, dwellings that had seasonal radon concentrations less than 3.7 Bq/m^3^, based on valid measures for the radon monitor, were excluded from the analysis and modeling. In all, 2518 of the 3893 dwellings and 633 of the 1154 dwellings surveyed by KINS and NIER, respectively, were used in this study. Table 1 provides a summary of the two surveys.

As shown in Table 1, indoor radon concentrations were highest in the winter and lowest in the summer. To compare ratios of concentrations in the winter and summer by region, we used an analysis of variance (ANOVA) and Tukey’s honest significant difference (HSD) test (R version 3.4.2). The ratios of winter to summer concentrations were significantly different among the regional groups (*p* < 0.001, ANOVA). However, we found little significance in dividing regions according the ratio of winter to summer concentrations (Tukey’s HSD). Meanwhile, the ratios of winter to summer concentrations were significantly different among residential types (*p* < 0.001, ANOVA). Moreover, results were significant according to detached houses and other types of residences using data from the KINS and NIER, respectively (*p* < 0.001, Tukey’s HSD). Overall, there was a significant difference between detached houses and other residences. Therefore, we derived seasonal correction factors according to housing type. Table 2 summarizes of the ratios of winter to summer concentrations of indoor radon according to housing type.

### 2.2. Seasonal Correction Factors

Predicted mean annual radon concentrations are calculated by multiplying the concentration measured during month j by the correction factor $s_{j}^{\left(t\right)}$ as follows:

(2) $$M^{\left(12\right)}=s_{j}^{\left(t\right)}M^{\left(t\right)}$$

where $M^{\left(12\right)}$ denotes a full 12 months of measurement results, $s_{j}^{\left(t\right)}$ denotes the seasonal correction factors, where j=1,…,12, and $M^{\left(t\right)}$ denotes the radon concentration measured during the t months, where t=1,…,12. In this formula, the seasonal correction factor $s_{j}^{\left(t\right)}$ is calculated as

(3) $$s_{j}^{\left(t\right)}=\frac{t}{12}\frac{\sum_{k=1}^{12}m_{k}}{\sum_{k=j}^{j+t-1}m_{k}}$$

where *t* denotes the measurement period, and $m_{k}$ denotes the integrated radon concentration in month *j*, where j=1,…,12 with the convention that $m_{k+12}=m_{k}$ [8]. The quantity $m_{k}$ might be interpreted as the geometric mean radon concentration in month k for the population of dwellings [8,9].

Since we only had four seasonal datasets, letting t=3 and j=1,4, 7, and 12, $\sum_{k=1}^{12}m_{k}/12$ might be interpreted as the annual geometric mean radon concentration, and $\sum_{k=j}^{j+2}m_{k}/3$ might be interpreted as the geometric mean radon concentration for each season in this study. Then, the seasonal correction factor for each season was derived. Furthermore, the seasonal adjustment factor $\hat{s_{j}^{\left(t\right)}}$ could also be obtained with the monthly adjusted radon concentration $\hat{m_{k}}$. In the next section, we present the seasonal variation model proposed by Pinel et al. [8].

### 2.3. Seasonal Adjustment Factor

Since indoor radon concentrations exhibit seasonal periodicity, we assumed that patterns of seasonal variations can be represented by the Fourier series as a linear combination of sine and cosine functions. Meanwhile, since we only had four seasonal datasets, we considered a simpler model:

(4) $$C_{j}=\alpha_{0}+\alpha_{1}\sin\left(\frac{j}{12}2\pi \right)+\alpha_{2}\cos\left(\frac{j}{12}2\pi \right)$$

where $C_{j}$ denotes the radon concentration in month j, and $\alpha_{0}$, $\alpha_{1}$, and $\alpha_{2}$ denote the Fourier coefficients.

Then, the geometric mean radon concentration in month j for the population of dwellings $m_{j}$ might be also represented by

(5) $$m_{j}=\alpha_{0}+\alpha_{1}\sin\left(\frac{j}{12}2\pi \right)+\alpha_{2}\cos\left(\frac{j}{12}2\pi \right)$$

It was not possible to apply this formula directly because the data in this study contained only 3-month measurements in four seasons rather than dividing into 12 months. Therefore, we obtained the Fourier coefficients $\alpha_{0}$, $\alpha_{1}$, and $\alpha_{2}$ based on the seasonal mean radon concentrations of the original data using the least-square regression method. 

**Figure 1:**
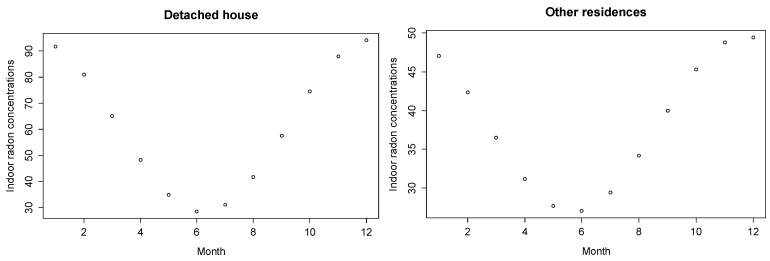
Monthly geometric mean of estimated indoor radon concentrations (Bq/m^3^) calculated from Equation (5) for different types of houses.

Since the pattern of variations assumed was sinusoidal, the monthly adjusted radon concentrations could be calculated using Equation (5). Figure 1 shows the monthly geometric mean of estimated indoor radon concentrations using Equation (5), and Table 3 shows the measured and estimated seasonal variations of indoor radon concentration for the different types of houses.

From Equation (5), together with estimates $\hat{m_{k}}$ from Table 3, the seasonal adjustment factors $\hat{s_{j}^{\left(t\right)}}$ could be obtained as follows:

(6) $$\hat{s_{j}^{\left(t\right)}}=\frac{t}{12}\frac{\sum_{k=1}^{12}\hat{m_{k}}}{\sum_{k=j}^{j+t-1}\hat{m_{k}}}$$

In Equation (6), letting t=3, and j=1,4, 7, and 12, the seasonal adjustment factor for each season could be derived.

### 2.4. New Correction Factors

The aim of this study was to divide seasonal correction factors for twelve separate month factors for general applicability. For this, we considered meteorological factors, such as outdoor temperature, as there were several studies about a correlation between indoor radon and outdoor temperature. Baysson et al. [13] and Krewski et al. [9] showed that indoor radon is correlated well with mean monthly outdoor temperatures. A study by Daraktchieva [12] also documented a strong negative correlation between geometric mean radon concentrations and mean monthly outdoor temperature (r = −0.99). However, a weak correlation between indoor radon and outdoor temperatures was noted under particular situations, such as being underground and a room that was unoccupied, by Denman et al. [10] and Groves-Kirkby et al. [17].

To investigate the correlation between indoor radon and outdoor temperature, we needed to obtain monthly radon concentrations. Since we had only datasets for four seasons, we estimated monthly indoor radon concentrations using Fourier series as mentioned in the previous section. In Equation (4), unlike previous sections, we used geometric mean and the log-transformed radon data, $\log C_{j}$ instead of arithmetic mean and $C_{j}$, respectively, for log-normal distribution of indoor radon concentrations. From the assumption of seasonal periodicity of indoor radon, the log-transformed radon data $\log C_{j}$ can be represented by

(7) $$\log C_{j}=\alpha_{0}+\alpha_{1}\sin\left(\frac{j}{12}2\pi \right)+\alpha_{2}\cos\left(\frac{j}{12}2\pi \right)$$

**Figure 2:**
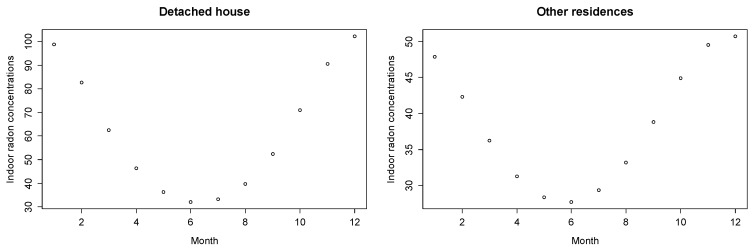
Monthly geometric mean of estimated indoor radon concentrations ($\mathrm{Bq}/m^{3}$) calculated from Equation (7) for different types of houses.

Figure 2 shows the monthly geometric mean of estimated indoor radon concentrations using Equation (7), and Table 4 shows the measured and predicted seasonal variations of indoor radon concentration in different types of houses.

**Figure 3:**
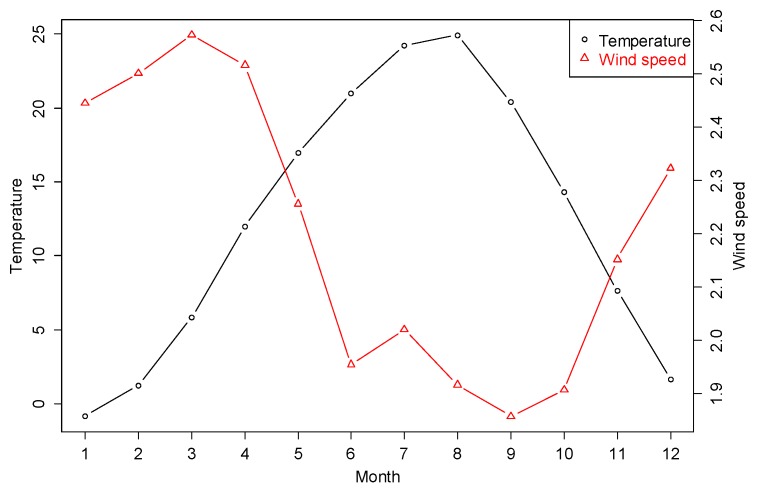
The monthly variations in outdoor temperature (°C) and wind speed ($m^{2}/s$).

Using the monthly geometric mean of estimated indoor radon concentrations obtained from Equation (7), indoor radon and outdoor temperature in Korea were negatively correlated for detached houses and other residences (r = −0.88 and r = −0.72, respectively): it was weaker than a noted correlation in the UK [12]. The result could be explained by radon patterns in the spring and fall. In detail, the highest concentrations of radon are found in the winter, followed by the fall, spring, and summer. Meanwhile, the highest outdoor temperatures in Korea are recorded in the summer, followed by the fall, spring, and winter (Figure 3).

To further explore correlations between indoor radon and meteorological factors other than outdoor temperature, we considered mean monthly wind speed, which was shown to be inversely correlated with indoor radon from the results of research by Miles [18]. For smoothness of year-to-year variations in mean monthly meteorological factors, 30-year averages (1981–2010) were derived from the National Climate Data Service System. Figure 3 shows the monthly variations in outdoor temperature and wind speed over 30 years.

The predicted mean annual radon concentration is calculated by multiplying the concentration observed during the month j of monitoring by the correction factors $f_{j}$ as follows:

(8) $$\log C_{\mathrm{year}}=f_{j}\log C_{j}$$

where $C_{\mathrm{year}}$ denotes the mean annual radon concentration, $C_{j}$ denotes the radon concentration during the month j, and $f_{j}$ denotes the correction factor, where j=1,…,12. As mentioned above, indoor radon is correlated with mean monthly outdoor temperature and wind speed [18]. Therefore, radon concentration during the month j is given in the following Equation:

(9) $$n_{j}=\beta_{0}+\beta_{1}d_{j}$$

where $n_{j}$ denotes the normalized monthly log radon concentration, and $d_{j}$ denotes the normalized meteorological factor associated with outdoor temperature and wind speed during the month j. We considered $d_{j}$ as follows:

(10) $$d_{j}=\frac{T_{j}T_{j+1}W_{j}}{\left|T_{j}T_{j+1}W_{j}\right|}$$

where $T_{j}$ denotes the mean outdoor temperature during the month j, with the convention $T_{j+12}=T_{j}$, and $W_{j}$ denotes the mean wind speed during the month j. With the normalized meteorological factor, the radon concentration was more strongly correlated with the meteorological factor $d_{j}$ than the mean outdoor temperature $T_{j}$ for detached houses and other residences (r = −0.96 and r = −0.88, respectively).

From Equations (8) and (9), the new correction factors are derived as follows:

(11) $$f_{j}=\frac{1}{\beta_{0}+\beta_{1}d_{j}}$$

With the new correction factor, mean annual radon concentration $C_{\mathrm{year}}$ is predicted as follows:

(12) $$C_{\mathrm{year}}=C_{j,k}^{\frac{f_{j}+\cdots +f_{j+k-1}}{k}}$$

where $C_{j,k}$ denotes the measured indoor radon concentration starting in j month during the k months.

## 3. Results and Discussion

### 3.1. Estimation of Seasonal Correction Factors

First, we estimated monthly radon concentrations from Equations (5) and (7), and calculated the correction factors based on Equations (3), (4) and (9). Table 5 shows the seasonal correction factors, seasonal adjustment factors, and new correction factors.

### 3.2. Applying the Seasonal Correction Factors to Data

Then, we estimated annual radon concentrations using the relevant correction factors and the observed seasonal radon concentrations for each season from two national surveys performed by the KINS and NIER. To evaluate the correction factors, we used the difference between estimated and measured mean annual radon concentrations: a negative difference means that the estimated value with a relevant correction factor is smaller than the measured value and vice versa.

**Figure 4:**
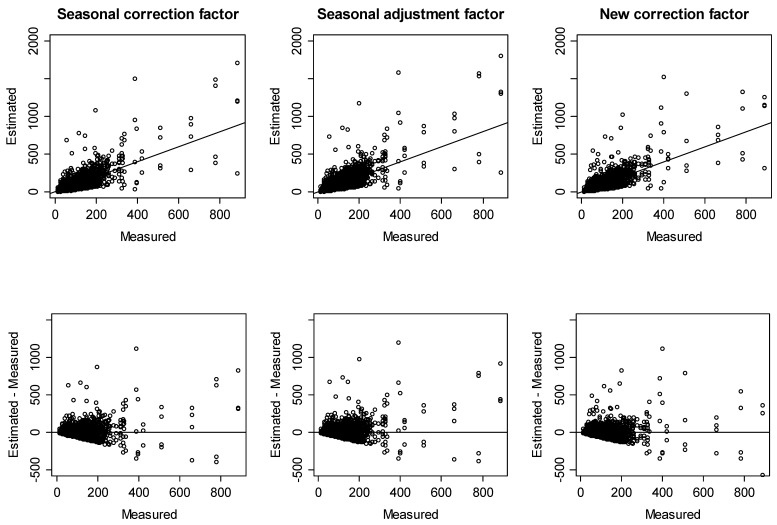
Comparisons of measured and estimated mean annual radon concentrations for detached houses.

**Figure 5:**
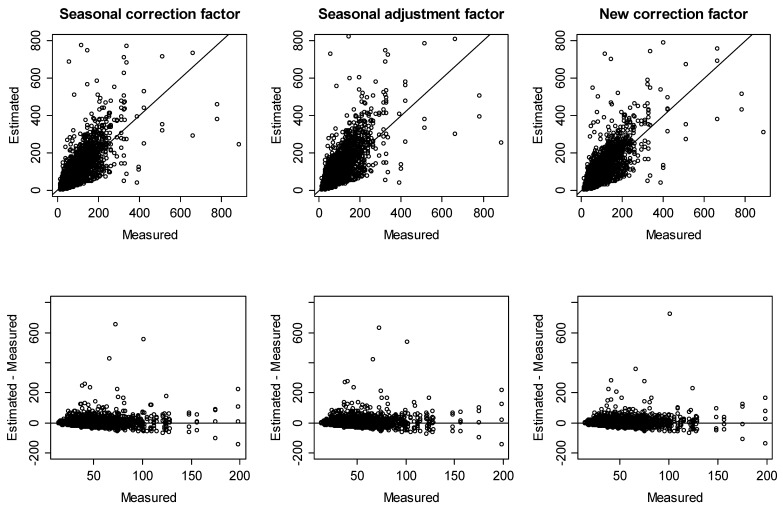
Comparisons of measured and estimated mean annual radon concentrations for other residences.

Figure 4 and Figure 5 show the concordance between measured and estimated mean annual radon concentrations for detached houses and other residences, respectively. 

To evaluate the accuracy of mean annual radon concentration predictions in more quantitative terms, we calculated the percentage of estimated mean annual radon concentrations with difference limits of ±10, ±20, ±30, and ±40 Bq/m^3^ and tested the equality of two proportions against the alternative that they are not equal by correction factors (Table 6). In detached houses, roughly 97% of the estimated values were within ±40 Bq/m^3^ of the measured value for all correction factors. In detail, although the seasonal correction factors had slightly better agreement than the new correction factors, except for within the difference limit of ±30 Bq/m^3^, there was no significant difference therein. Meanwhile, however, there was a significant difference in that the new correction factors had better agreement than the seasonal adjustment factors. Thus, we could predict the mean annual radon concentration for detached houses using Equation (2) and use the seasonal correction factors or the new correction factors when indoor radon concentrations are measured for a specific season. Otherwise, we can use Equation (12) and the new correction factors. 

For other residences, roughly 85–87% of the estimated values were within ±40 Bq/m^3^ of the measured value for all correction factors. Specifically, there was a significant difference in that the seasonal correction factors and the seasonal adjustment factors had better agreement than the new correction factors within the difference limit of ±10 Bq/m^3^. However, there was no significant difference in agreement between the seasonal correction factors and the seasonal adjustment factors, compared to the new correction factors, within the difference limit of ±20 Bq/m^3^ or more. Thus, we could predict the mean annual radon concentration for other residences using Equation (2) and the seasonal correction factors or seasonal adjustment factors when indoor radon concentrations are measured for a specific season. Otherwise, we can use Equation (12) and the new correction factors.

### 3.3. The Annual Effective Dose Model

In the UNSCEAR (2000) report [6], the annual effective dose by radon is calculated as follows:

(13) E=Q×F×T×K

where *Q* is the mean annual radon concentration, *F* is the equilibrium factor between indoor radon and its decay product, *T* is the annual residential time, and *K* is the dose conversion coefficient. Since 2000, the equilibrium factor for indoor and the dose conversion coefficient have been proposed 0.4 and 9 nSV/(Bq⋅h)/m^3^, respectively [6]. The information about the equilibrium factor for indoor and the dose conversion coefficient before 2000 was presented in the UNSCEAR (2000) report [6]. Since we can predict mean annual radon concentrations with measured indoor radon concentrations, the annual effective dose can be assessed using Equation (13) with the estimated mean annual radon concentration.

## 4. Conclusions

In this study, we investigated the predictability of mean annual radon concentrations based on data from surveys by KINS and NIER. Indoor radon concentrations in Korea were highest in the winter and lowest in the summer. Meanwhile, the ratio of winter to summer concentrations differed significantly between detached house and other types of residences. Therefore, we derived the seasonal correction factors, seasonal adjustment factors, and the new correction factors according to housing types. First, we derived seasonal correction and seasonal adjustment factors for each season with estimated monthly radon concentrations using the seasonal variation model based on Fourier series. Second, we investigated correlations between indoor radon and meteorological factors, such as outdoor temperature and wind speed. From the seasonal correction factors for each season and the estimated monthly radon concentrations, we extrapolated the new correction factors for each month.

To evaluate the correction factors, we assessed differences between estimated and measured mean annual radon concentrations. The results indicated that the seasonal correction factors and the new correction factors performed consistently better on the selected datasets than the seasonal adjustment factors. To evaluate the accuracy of mean annual radon concentration predictions in more quantitative terms, we calculated the percentage of estimated mean annual radon concentrations with differences limits of ±10, ±20, ±30, and ±40 Bq/m^3^ and tested the equality of two proportions against the alternative that they are not equal by correction factors. Upon doing so, we found that roughly 97% of the estimated values were within ±40 Bq/m^3^ of the measured value for detached houses; meanwhile, roughly 85–87% of the estimated values were within ±40 Bq/m^3^ of the measured value for other residences. For predicting mean annual radon concentrations for detached houses, we suggest that the seasonal correction factors or new correction factors can be of use when actual measurements of indoor radon concentrations for a specific season are available. Meanwhile, for predicting mean annual radon concentrations for other residences, it is possible to predict mean annual radon concentrations using either one when indoor radon concentrations are measured for a specific season. Unless measured in a specific season, the new correction factors may be more readily applicable.

Since we derived the correction factors according to averages, this study has a limitation in that there were large differences between estimated and measured mean annual radon concentrations in some houses. In other words, the correction factor may not show good performance in some houses with very large or very small seasonal variations. Nonetheless, the new correction factors can be reasonably used to predict mean annual radon concentrations if measurements of actual indoor radon concentrations are available for any period of time. Furthermore, they may be of use for more accurate assessment of annual effective doses.

## Figures and Tables

**Table 1:** Indoor radon concentrations in the Korea Institute of Nuclear Safety (KINS) and National Institute of Environmental Research (NIER) surveys for each season.

	Seasonal Radon Concentration (Bq/m^3^)									
	N	AM	SD	GM	GSD	Min	Q_1_	Median	Q_3_	Max
KINS (2005)										
Spring	2518	53.9	55.5	41.8	1.9	8.5	25.9	40.7	62.9	1073.0
Summer	2518	32.8	26.9	28.3	1.6	6.0	18.5	26.5	37.0	558.7
Fall	2518	69.5	78.1	55.2	1.9	11.1	37.0	51.8	77.7	1835.0
Winter	2518	86.1	135.3	60.5	2.1	4.0	37.0	55.5	92.5	3249.0
NIER (2010–2011)										
Spring	633	53.9	49.7	43.0	1.9	5.7	27.4	40.8	61.4	651.2
Summer	633	47.9	26.5	43.2	1.5	9.4	32.6	42.0	54.9	333.2
Fall	633	107.4	93.9	86.4	1.8	20.0	57.3	77.0	119.0	1172.0
Winter	633	124.1	135.9	88.1	2.2	7.1	50.5	77.8	140.0	1509.0

Abbreviations: AM, arithmetic mean; SD, standard deviation; GM, geometric mean; GSD, geometric standard deviation; Q_1_, first quartile; Q_3,_ third quartile; KINS, Korea Institute of Nuclear Safety; NIER, National Institute of Environmental Research.

**Table 2:** Winter/summer ratios of indoor radon concentrations in different types of houses.

		Winter/Summer Ratio									
		KINS			NIER				Total		
		N	AM	GM	N	AM		GM	N	AM	GM
Detached house	Korean-style	1347	3.59	2.74	117		3.02	2.18	1761	3.57	2.70
	Western-style				297		3.68	2.75			
Other residences	Apartment	961	1.83	1.55	106		1.29	1.14	1390	1.85	1.56
	Row house	210	2.28	1.91	84		1.61	1.46			
	Multiplex house				29		1.83	1.60			

Abbreviations: KINS, Korea Institute of Nuclear Safety; NIER, National Institute of Environmental Research.

**Table 3:** Measured and estimated seasonal variations in indoor radon concentration calculated from Equation (5) for different types of houses.

		Measured (Bq/m^3^)	Estimated (Bq/m^3^)	Percent Error ^a^ (%_error_)
Detached house	Spring	50.60	49.39	2.39
	Summer	32.52	33.73	−3.73
	Fall	74.56	73.35	1.62
	Winter	87.80	89.01	−1.38
Other residences	Spring	33.26	31.76	4.52
	Summer	28.69	30.19	−5.24
	Fall	46.21	44.71	3.25
	Winter	44.77	46.27	−3.36

^a^ (Measured−Estimated) × 100/Measured.

**Table 4:** Measured and predicted seasonal variations in indoor radon concentration calculated from Equation (7) for different types of houses.

		Measured (Bq/m^3^)	Estimated (Bq/m^3^)	Percent Error ^a^ (%_error_)
Detached house	Spring	50.60	47.19	4.52
	Summer	32.52	34.86	−5.24
	Fall	74.56	69.54	3.25
	Winter	87.80	94.13	−3.36
Other residences	Spring	33.26	31.80	4.39
	Summer	28.69	30.01	−4.59
	Fall	46.21	44.19	4.39
	Winter	44.77	46.83	−4.59

^a^ (Measured−Estimated) × 100/Measured.

**Table 5:** Correction factors for detached houses and others residences.

Season	Month	Detached House			Other Residences		
		$f_{j}\;{\;}^{a}$	$s_{j}^{\left(3\right)}{\;}^{b}$	$\hat{s_{j}^{\left(3\right)}}{\;}^{c}$	$f_{j}{\;}^{a}$	$s_{j}^{\left(3\right)}{\;}^{b}$	$\hat{s_{j}^{\left(3\right)}}{\;}^{c}$
**Spring**	March	0.94	1.13	1.24	0.97	1.13	1.20
	April	1.01			1.01		
	May	1.09			1.04		
**Summer**	June	1.14	1.76	1.82	1.07	1.31	1.27
	July	1.21			1.10		
	August	1.13			1.07		
**Fall**	September	1.02	0.77	0.84	1.01	0.81	0.86
	October	0.95			0.97		
	November	0.91			0.95		
**Winter**	December	0.90	0.65	0.69	0.95	0.83	0.83
	January	0.91			0.95		
	February	0.91			0.95		

^a^ New correction factor. ^b^ Seasonal correction factor. ^c^ Seasonal adjustment factor.

**Table 6:** Percentage of estimated mean annual radon concentrations within specific difference limits according to types of houses and correction factors.

		N	Difference Limit (Bq/m^3^), *p*-Value							
			±10	*p*-Value	±20	*p*-Value	±30	*p*-Value	±40	*p*-Value
Detached house	Seasonal correction factor	7044	45.5	0.498	70.6	0.618	81.4	0.879	87.3	1.000
	Seasonal adjustment factor	7044	43.2	0.038	67.1	0.000	78.5	0.000	85.3	0.001
	New correction factor	7044	44.9	—	70.2	—	81.5	—	87.3	—
Other residences	Seasonal correction factor	5560	65.7	0.001	88.9	0.062	95.1	0.760	97.2	0.727
	Seasonal adjustment factor	5560	64.7	0.020	88.5	0.265	94.8	0.670	97.0	0.278
	New correction factor	5560	62.6	—	87.8	—	95.0	—	97.3	—
